# Carrier Dynamic Investigations of AlGaInAs Quantum Well Revealed by Temperature-Dependent Time-Resolved Photoluminescence

**DOI:** 10.3390/ma13194227

**Published:** 2020-09-23

**Authors:** Yue Song, Yongyi Chen, Ligong Zhang, Yugang Zeng, Cheng Qiu, Lei Liang, Yuxin Lei, Peng Jia, Li Qin, Yongqiang Ning, Lijun Wang

**Affiliations:** 1 State Key Laboratory of Luminescence and Applications, Changchun Institute of Optics, Fine Mechanics and Physics, Chinese Academy of Sciences, Changchun 130033, China; songyue@ciomp.ac.cn (Y.S.); qiucheng@ciomp.ac.cn (C.Q.); liangl@ciomp.ac.cn (L.L.); leiyuxin@ciomp.ac.cn (Y.L.); jiapeng@ciomp.ac.cn (P.J.); qinli@ciomp.ac.cn (L.Q.); ningyq@ciomp.ac.cn (Y.N.); wanglj@ciomp.ac.cn (L.W.); 2 Center of Materials Science and Optoelectronics Engineering, University of Chinese Academy of Sciences, Beijing 100049, China; 3 Peng Cheng Laboratory, No. 2, Xingke 1st Street, Shenzhen 518000, China

**Keywords:** AlGaInAs quantum well, time-resolved PL, carrier dynamic, decay time

## Abstract

AlGaInAs quantum well (QW) lasers have great potential in the application fields of optical communications and eye-safety lidars, owing to the advantages of good gain performance. A large amount of experimental evidence indicated that carrier dynamic affects the resonant frequency and modulation response performance of QW lasers. However, the mechanism of carrier dynamic in AlGaInAs QW structure is still ambiguous for complicated artificial multilayers. In this paper, the carrier dynamic of AlGaInAs QW structure was investigated by temperature-dependent time-resolved photoluminescence (TRPL) in the range of 14 to 300 K. Two relaxation times (a fast component and a slow one) have a major impact on the PL emission spectra of the AlGaInAs QW below 200 K. The carriers prefer a fast decay channel in the low temperature regime, whereas the slow one a higher temperature. An unconventional temperature dependence of carrier relaxation is observed in both decay processes. The carriers’ lifetime decreases with the temperature increasing till 45 K and then increases with temperature up to 250 K. It is quite different from that in the bulk semiconductor. The mechanism of temperature-dependent carrier relaxation at temperatures above 45 K is a combination of dark state occupation and a nonradiative recombination process.

## 1. Introduction

1.3–1.5 μm wavelength semiconductor quantum well (QW) lasers have great potential in the application fields of optical communications, eye-safety lidar and three-dimensional imaging [1,2]. Due to the large conduction-band offset and low valence-band offset, AlGaInAs series of III–V materials that take advantage of good high-temperature performance, suitable photon energy and high material gain have become indispensable for 1.3–1.5 μm InP-based laser diode [3,4,5]. AlGaInAs QW lasers exhibit higher modulation-speed, more superior high-temperature performance than the frequently-used InGaAsP/InP lasers [6,7,8]. However, the epitaxial growth process of the quaternary AlGaInAs QW structure is very complex, leading to some complicated localized states produced in the active region. These states strongly affect the emission transition of carriers in the AlGaInAs QW.

Experimental reports and theoretical analysis indicate that the capture and escape rate of carriers affect both the resonant frequency and damping rate of the modulation response for QW laser diodes [9,10,11,12]. However, the mechanism of such effect is still unclear for the quaternary AlGaInAs QW. The carrier dynamic process of the AlGaInAs QW structure has been intriguing researchers for a few decades. The dynamic characteristics of AlGaInAs/InP microlasers subject to optical injection were reviewed by Y.Z. Huang, et al. [13]. PL decay of the thermal-treated AlGaInAs QW demonstrated that the effect of thermal process had a strong influence on the relaxation time of excitated carriers [14].

Moreover, an S-type behavior in temperature-dependent PL peak energy was observed in quaternary AlGaInAs QW and some ternary III–V group semiconductors in the low temperature regime. It was reported that the behavior in AlGaInAs is remarkable in comparison to many ternary III–V group semiconductor materials [15,16]. Researchers have not reached an agreement on the mechanism of such behavior [17,18]. In general, there are two theories to model the S-type behavior in the ternary materials systems. One is based on the carriers’ capturing and thermally escaping process from localized centers [19]. The other is based on the recombination processes of localization and delocalization [20]. Neither of the two models could explain the significant S-type behavior for the quaternary AlGaInAs QW structure. The dynamics analysis of carriers at the emission center could help to give some insight into the changes of energy level.

In our previous work, we investigated the mechanism of temperature-dependent PL peak energy of AlGaInAs QWs [14]. The dependencies and relationships between PL peak energy and FWHM vs. temperatures were discussed. In this paper, we attempted to study the mechanism of temperature-dependent carrier dynamic in the AlGaInAs QW structure. TRPL is commonly regarded as a powerful tool for detecting the dynamic behavior of carriers in a direct-gap semiconductor [9]. As temperature is crucial to the transition rate of electrons and holes, especially for those transporting to nonradiative recombination centers, we mainly focus our attention on temperature dependence of such dynamics in this paper. Temperature-dependent TRPL is applied to characterize the spontaneous emission process of the AlGaInAs QW in the temperature range from 14 K to room temperature. The influence of the carriers’ dynamic process on the optical properties and localization of carriers are also discussed. The study of carrier relaxation time is very helpful for improving the modulation rate of AlGaInAs based devices.

## 2. Materials and Methods

**Figure 1:**
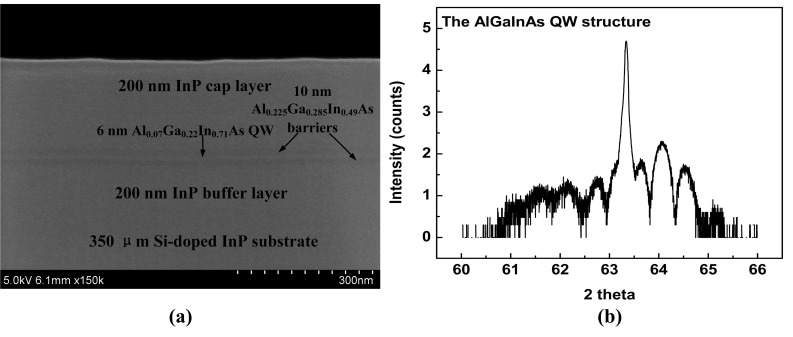
(**a**) Cross-sectional SEM image of the AlGaInAs quantum well (QW) structure. (**b**) Intensity (counts)-2θ scans of HR-XRD pattern.

The AlGaInAs QW structure was grown via a Metal Organic Chemical Vapor Deposition system (AIXTRON RF 200/4; Herzogenrath, Germany) on Si-doped InP substrate oriented (001). A 6 nm Al_0.07_Ga_0.22_In_0.71_As QW sandwiched between two 10 nm Al_0.225_Ga_0.285_In_0.49_ As barriers, was embedded in a 200 nm InP cap layer and a 200 nm InP buffer layer. Figure 1 shows the cross-sectional scanning electron microscope (SEM; Hitachi, Tokyo, Japan) and high-resolution x-ray diffraction (HR-XRD; Bruker, Billerica, MA, USA) of the AlGaInAs QW structure. As seen in Figure 1a, the interfacial morphologies between different epitaxial layers are quite well. The X-ray diffraction pattern of the AlGaInAs QW structure is composed of a dominated InP diffraction peak localized at 63.345 °C (2 theta) and the QW’s multiorder satellite peaks at the side vicinity. More details about the structure property and growth method were presented in our previous work [14]. The AlGaInAs QW was measured by the temperature-dependent TRPL (HJY TRIX550; Original, USA). A cryogenic holder (ARS 8200 cryogenic system; ARS, USA) was applied to stabilize the temperature of a sample in the range of 14–300 K. PL decay time was detected on a fluorescence spectrometer FL920 (Edinburgh Instruments Ltd., Livingston, UK) equipped with a time correlated single-photon counting technique, accompanied by a Hamamatsu R5509-72 fast time respond infrared photomultiplier (PMT; Hamamatsu Photonics, Hamamatsu, Japan) in liquid Nitrogen with a response time of 800 ps. PL emission was monitored at the maximum emission wavelength under an excitation of a 635-nm picosecond pulse diode laser with a pulse duration of 180 ps and a repetition frequency of 1 MHz. The TRPL at a certain temperature was obtained by assembling a series of fluorescent decay curves, which were detected in a group of wavelengths with an interval of 5 nm in wavelengths from 1280 to 1520 nm and with an optimized detecting time constant.

## 3. Results

**Figure 2:**
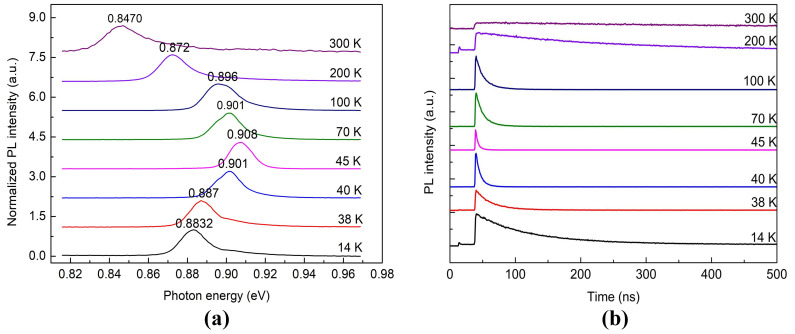
(**a**) Normalized steady-state PL spectra. (**b**) PL decay curves of AlGaInAs QW at different temperatures.

PL spectrum of AlGaInAs QW presents an emission band corresponding to a 6 nm Al_0.07_Ga_0.22_In_0.71_ As active layer at room temperature. The central peak energy at room temperature was 0.847 eV, which is in accordance with the theoretical data [21,22,23,24]. The steady-state PL spectra for the emission band of AlGaInAs QW at various temperatures from 14 to 300 K are shown in Figure 2a. All of the PL spectra revealed an asymmetric emission spectrum with a trailing edge on the high energy side. An abnormal nonmonotonic change on the radiative recombination energy of AlGaInAs QW is observed. As the temperature decreased from room temperature, the emission peak tended to shift to higher energy and reached the maximum point at around 45 K. However, when the temperature decreased below 45 K, the trend was inverted [25]. Nontrivial temperature dependency on carrier relaxation was also observed. The series of emission decay traces at different temperatures for the AlGaInAs QW is shown in Figure 2b, while the detection wavelengths were located at the emission peak of each spectrum for a certain temperature. The PL decay curves followed a single-exponential function in the range of 200 K < T < 300 K, indicating that there was only a single-emission center transition process in each emission spectrum. While temperature was below 200 K, the fluorescence decay behaviors appeared to evolve into a double-exponential function decay process in the low temperature regime. The fluorescent decay consisted of a fast decay component and a slow decay component, reflecting that multicentral excited states contributed to the observed PL emission of the AlGaInAs QW.

As an attempt to obtain insight into the evolution of the transient spectra and overall emission spectrum, TRPL was introduced at this stage.

**Figure 3:**
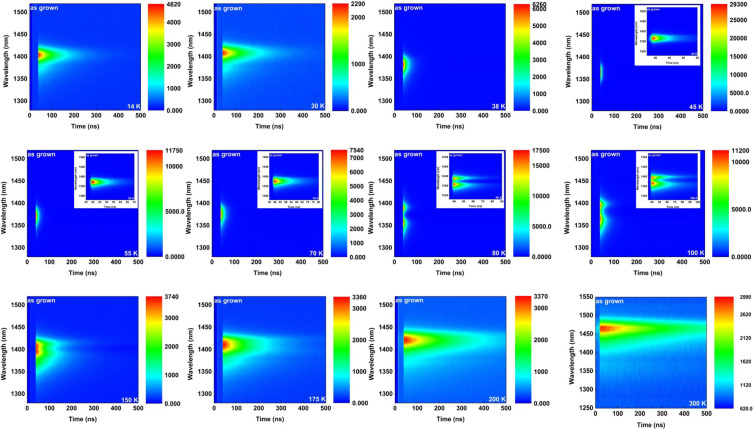
Overview of the time-resolved PL data for AlGaInAs QW.

The processes of carriers’ transfer, recombination and trapping among different excited-state or center and ground-state in QW/superlattice structure could be investigated by TRPL. An overview of TRPL for the AlGaInAs QW in a temperature interval from 14 to 300 K is shown in Figure 3. Zoom-in plots were inserted in the temperature range from 45 to 100 K. The color bar at the right-hand side of each map reflects the numerical value of PL intensity. As seen in Figure 3, as the experimental temperature increased, an obvious increment in PL intensity can be observed. The intensity reached its local maximum intensity at around 45 K. Furthermore, the intensity of AlGaInAs QW showed a downward trend, which is in good agreement with our previous work [25].

As shown in Figure 3, the transient PL spectra revealed two peaks in the emission band below 200 K. It can be obviously observed that there were two intensity-equilibrated discrete emission bands in the temperature interval from 45 to 175 K. For most of the other temperatures, a main narrow emission spectrum peak on the low-energy side is superposed with a broad-bandwidth emission spectrum peak on the high-energy side. The superposition attenuates as the temperature increase.

**Figure 4:**
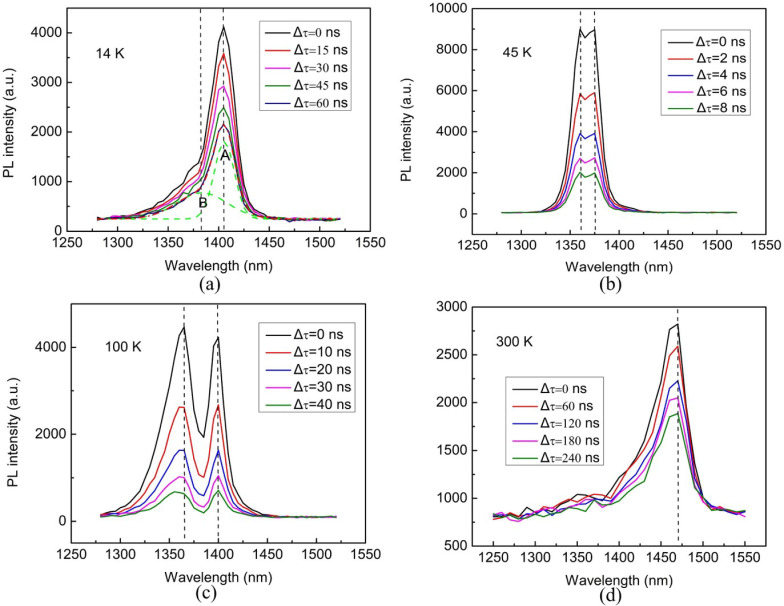
TRPL spectra measured at (**a**) 14 K, (**b**) 45 K, (**c**) 100 K and (**d**) 300 K at various delay times.

In order to investigate the dynamic behavior of the emission centers, PL spectra at 14, 45, 100 and 300 K with several specified time delay after pulse excitation are plotted in Figure 4. The asymmetric emission band profile at 14 K can be well fitted using a sum of two Gauss functions, shown as a short-dashed green line in Figure 4a. Based on the double-peak emission simulation, we found that the spectrum profile and central position of the emission peak for the two emission spectra did not change with the time elapsed. This indicates that the recombination rate in each emission band is time-invariant, and the relaxation times for wavelengths distribution in emission band are generally consistent. This is a confirmation that the emission should be originated from two certain emission centers.

For many material systems, as the time elapsed, the transient PL spectra shifted towards the lower energy side accompanied with a sharp drop in PL intensity due to the carriers trapping by localized states at low temperatures [26,27]. In our case, we did not observe any shift in PL peak by increasing the delay time. Therefore, the effects of carrier localization are not obvious in the AlGaInAs QW structure at low temperatures.

It also shows from TRPL that the emission bands of the two emission centers overlapped strongly at low temperatures (<80 K). As temperature increased, the two emission bands appeared to separate. In order to distinguish the decay characteristics of excitation states for the two emission centers, we fitted the PL decay curve at a certain wavelength in an emission band by double exponential decay functions, containing a fast-decay part and a slow-decay part [28].

(1) $$n\left(t\right)=a_{\mathrm{slow}}\times e^{-t/\tau_{slow}}+a_{\mathrm{fast}}\times e^{-t/\tau_{fast}}$$

where a_slow_ and a_fast_ represent the amplitude of the slow and the fast decay component. *τ_slow_* and *τ_fast_* are the carrier recombination lifetime of the two components. The proportion of slow decay component is calculated by a_slow_·*τ_slow_*/(a_slow_·*τ_slow_* + a_fast_·*τ_fast_*) and the proportion of fast decay component is a_fast_·*τ_fast_*/(a_slow_·*τ_slow_* + a_fast_·*τ_fast_*).

**Figure 5:**
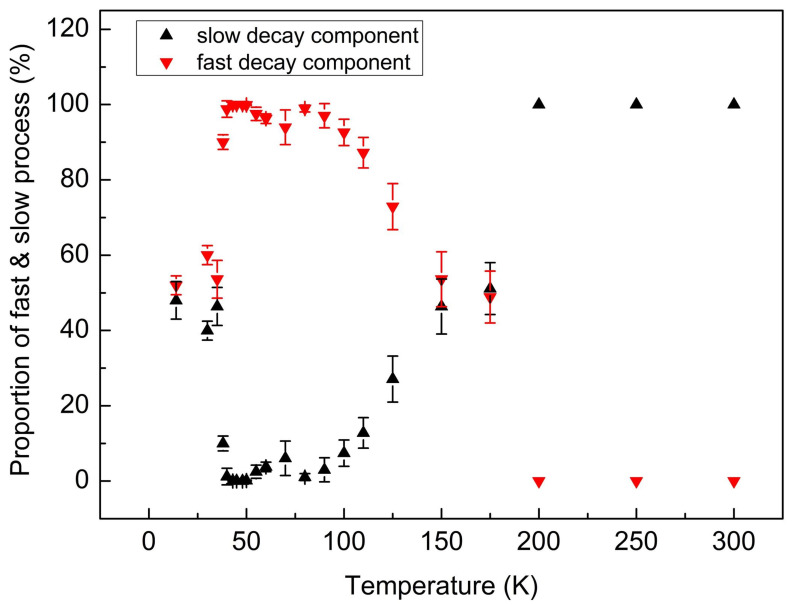
The proportion of slow and fast decay component detected at the maximum emission peak as functions of temperature.

Figure 5 shows the proportion of the slow decay component and the fast decay component for the main emission peak at different temperatures. The error bar in Figure 5 represents the major proportion of fast/slow decay component distribution of the luminescence decay times measured at different wavelengths in an interval of 20 nm centered at the main emission peak. It is indistinguishable owing to the large overlap between the two emission spectra, the data shown here summon both the carrier lifetimes of the broad-bandwidth peak and the main narrow emission peak. Below 38 K, the contribution of the fast and slow components to the decay phase was almost 50%, respectively. In the temperature interval of 38–125 K, the decay curve was dominated by the fast-decay component, meaning that more carriers selected the fast decay channel. When temperature was elevated to 175 K, the ratio of fast and slow decay constants converged to one again. Above 175 K, the fast-component disappeared because of the thermally activated nonradiative process. Therefore, the single-centered emission transition process in the high temperature range is attributed to the slow decay component. The fast decay component is corresponding to the broad-bandwidth emission peak on the high-energy side, and the slow one corresponding to the main emission band on the low-energy side.

**Figure 6:**
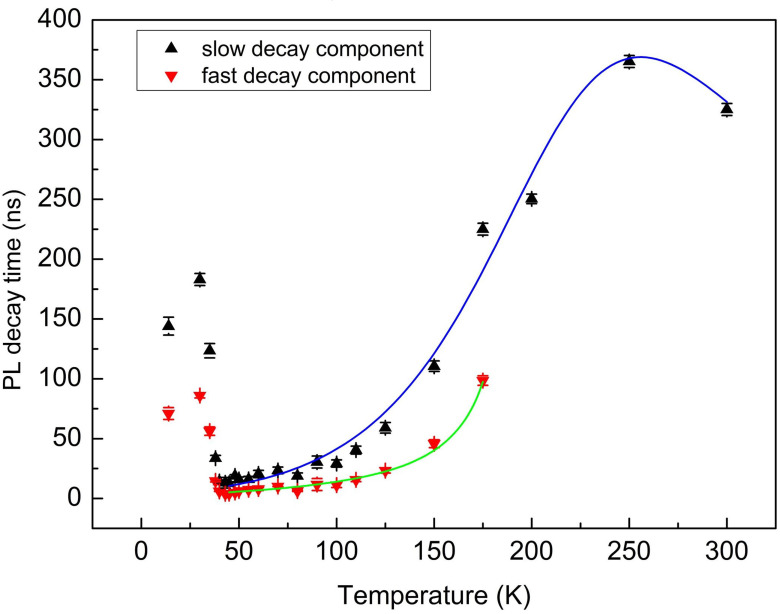
Temperature dependence of PL decay lifetimes of the AlGaInAs QW structure.

In general, it is difficult for thermally activated nonradiative processes to occur in the low-temperature regime [27]. Additionally, as the interaction between electron and phonon was suppressed at low temperatures, the carrier lifetime of the maximum main emission wavelength at 14 K was relatively long. The PL decay time decreased rapidly with the temperature increasing due to the weaken inhibition effect of the nonradiative recombination process. However, it reached the minimum at 45 K; then, when the temperature continued to rise, the decay time increased slowly until 250 K. The evolution of PL decay-times with temperature is plotted in Figure 6.

Figure 6 depicts the PL decay time constants of AlGaInAs QW as a function of temperature. The error bar in Figure 6 represents a 95% significant distribution of luminescence lifetimes measured at different spectral positions in an interval of 20 nm centered at the main emission peak. Double-central transitions had a major impact on the PL emission of AlGaInAs QW in the temperature range below 200 K. The carrier escape process exhibited a pronounced nonmonotone change for the fast and slow decay components. The escape rate of carriers in AlGaInAs QW increased with the temperature increasing to 45 K, and then decreased, unlike the carrier decay time dropping with temperature elevating due to the process of nonradiative recombination reported in most bulk semiconductors [29]. At the temperature below 45 K, the decay time constant of the slow-component showed a noticeable decreasing trend from 144 to 12.6 ns with temperature rising up to 45 K, while the decay time constant of the fast-component decreased slowly from 70.9 to 4 ns. In the middle temperature range of 45–100 K, the differences in decay time constants between the fast and slow-component became smaller (less than 18 ns). Then, the PL decay time of AlGaInAs QW became longer with the increase in temperature and reached 225 and 98.4 ns at 175 K. At higher temperature above 175 K, only one decay process was observed.

The increase in carrier lifetime in the range above 45 K is inconsistent with the temperature dependence of the radiative recombination process in most bulk semiconductor materials [30,31]. For InAs/GaAs nanostructures, C.H. Lin attributed it to the existence of another carrier compensation channel, which was normally caused by the recombination of dark states (states that can neither be accessed by absorbing photons or relax to other energy states nonradiatively) [32]. B.S. Eller proposed a temperature-dependent decay time mechanism in which the presence of the polarization-field in the samples led to the change of surface band bending, and resulted in a reduction in the overlap between the electron and hole wave functions [33]. The separation gave rise to a decrement in exciton oscillator strength, and a longer decay time at a high temperature [34]. However, the polarization-field strain should simultaneously affect the position of the emission peak, bandwidth and intensity. We tend to attribute it to the dark state model.

We attempted to apply a dark state model [31,32] to our case above 45 K. The equation is listed below:

(2) $$1/\tau =1/\tau_{0}-\exp(-∆E_{1}/KT)/\tau_{1}$$

where *τ*_0_ represents the lifetime of carriers at the ground state at 0 K, Δ*E*_1_ indicates the energy difference between the dark state and the ground state, K is the Boltzmann constant 8.6173 × 10^−5^ eV/K and *τ*_1_ is the decay time from dark state to the ground state. The optimizing parameter *τ*_0_ from the best fit of experimental data for the fast decay component is 2.67 ns. The parameter Δ*E*_1_ value is 3.5 meV. The relaxation time between the dark state and ground state *τ*_1_ is 2.17 ns.

As can be seen in Figure 6, the increasing trend in fast-component can be well fitted by the model above (shown as solid green line). It is suggested that the increase in PL decay time in the fast-component was caused by the occupation of dark state.

However, the model above cannot simulate the experimental data of the slow decay component, which governs the recombination process of carriers at a higher temperature. We propose a new model based on the dark state equation, which is amended by a nonradiative item to fit the experimental data of the slow-component. The fitting curve is drawn in the solid blue line in Figure 6.

(3) $$1/\tau =1/\tau_{0}-\exp(-∆E_{1}/KT)/\tau_{1}+\exp(-∆E_{2}/KT)/\tau_{2}$$

Δ*E*_2_ indicates the thermal activation energy, and *τ*_2_ represents the nonradiative lifetime.

Here, the optimizing parameters *τ*_0_, Δ*E*_1_, *τ*_1_, Δ*E*_2_ and *τ*_2_ for slow decay component are 6.94 ns, 6.3 meV, 3.08 ns, 15.1 meV and 4.91 ns, respectively. It fits quite well in the whole temperature region from 45 to 300 K. It is demonstrated that the dramatic increase in PL decay at temperatures above 45 K is affected by the combined effect of both dark states and carriers’ nonradiative recombination process. At present, the origin of dark states in the AlGaInAs QW structure is still ambiguous; more experiments are required to clarify the issue. The further explanation of the dark states will be discussed in our future work.

## 4. Conclusions

The carrier dynamic process of AlGaInAs QW structure is investigated systematically by temperature-dependent TRPL in the temperature interval from 14 to 300 K. Double-central transitions were observed at temperatures below 200 K. It indicates that the carriers prefer a fast decay channel in the low temperature regime, whereas the slow one at a higher temperature. The fast decay component reflects the broad-bandwidth emission peak on the high-energy side, while the slow decay component corresponds to the main emission band on the low-energy side. The carrier escape processes exhibit a nonmonotone change for both decay processes. The escape rate of carriers in AlGaInAs QW increases with the temperature increasing to 45 K, and then decreases with temperature up to 250 K. The carrier localization is not obvious at low temperatures in the quaternary AlGaInAs QW. The temperature-dependent carrier relaxation at temperatures above 45 K is attributed to the combined effect of dark state occupation and the nonradiative recombination process.
